# Bacterial pericarditis after endobronchial ultrasound-guided transbronchial needle aspiration

**DOI:** 10.1016/j.rmcr.2026.102411

**Published:** 2026-03-23

**Authors:** Rachna S. Gulati, Håkon Kravdal, Heidi Hoven, Erlend Eriksen, Hans Kristian Haugland, Tehmina Mustafa

**Affiliations:** a Department of Thoracic Medicine, Haukeland University Hospital, Bergen, Norway; b Department of Microbiology, Haukeland University Hospital, Bergen, Norway; c Department of Radiology, Haukeland University Hospital, Bergen, Norway; d Department of Cardiology, Haukeland University Hospital, Bergen, Norway; e Department of Pathology, Haukeland University Hospital, Bergen, Norway; f Center for International Health, Department of Global Public Health and Primary Care, University of Bergen, Norway

**Keywords:** Endobronchial ultrasound, Transbronchial needle aspiration, Bronchoscopy, Bacterial pericarditis, Lung cancer

## Abstract

**Background:**

Endobronchial ultrasound-guided transbronchial needle aspiration (EBUS-TBNA) is a valuable diagnostic and staging tool for lung cancer and is generally considered a safe procedure with very low complication rates. Bacterial pericarditis is a rare but serious complication following EBUS-TBNA. Here we describe a case of bacterial pericarditis that developed after an EBUS-TBNA procedure.

**Case summary:**

A 61-year-old woman underwent EBUS-TBNA to investigate a tumor in her right lung. Two weeks after the procedure she presented to the emergency department due to dizziness, nausea and chest pain. Her condition rapidly deteriorated, leading to hypotension and cardiogenic shock. She was diagnosed with bacterial pericarditis resulting in cardiac tamponade. Emergency pericardial drainage was performed and treatment with antibiotics was started along with adjunctive corticosteroids. Although she initially responded well to treatment, she later developed constrictive pericarditis, which caused a slight reduction in cardiac output. She also responded well to treatment with Osimertinib for non-small-cell lung cancer.

**Conclusion:**

Bacterial pericarditis following EBUS-TBNA is a rare but severe complication. It has been associated with certain risk factors, particularly sampling necrotic lesions near the pericardium.

## 1 Introduction

Endobronchial ultrasound-guided transbronchial needle aspiration (EBUS-TBNA) is a minimally invasive technique used to visualize structures within the airway wall, lungs and mediastinum. Its primary role is in the diagnosis and staging of lung cancer, but it is also used for sampling mediastinal lymphadenopathy, central masses, nodules and peribronchial lesions [1].

EBUS-TBNA is generally considered safe with complications rates ranging from 0.08 to 6.8% [2]. The most common complications include hypotension, hypoxia, bleeding and pneumothorax, while less frequent but more serious complications involve cardiorespiratory arrest, respiratory failure and airway perforation [2]. Bacterial pericarditis is a rare complication after EBUS-TBNA with only a few reported cases [3].

In this case report, we present a severe instance of bacterial pericarditis leading to cardiac tamponade and acute respiratory distress syndrome (ARDS) as a complication following EBUS-TBNA. Despite treatment with pericardial drainage, intravenous antibiotics and corticosteroid therapy, the patient developed constrictive pericarditis.

## 2 Case

A 61-year-old woman with a history of smoking presented with acute position-dependent vertigo and was initially diagnosed with benign paroxysmal position-dependent vertigo. As her symptoms worsened, magnetic resonance imaging (MRI) eventually revealed multiple brain metastases, for which she received symptomatic treatment with dexamethasone.

Computed tomography (CT) scan of the thorax and abdomen revealed a tumour in the right hilum, multiple enlarged mediastinal lymph nodes corresponding to stations 2, 4R, and 7 as well as liver metastases. The tumour occluded the middle lobe bronchus and invaded the left atrium.

Bronchoscopy and EBUS-TBNA were performed five days later. Bronchoscopy revealed tumorous changes at the entrance to the right middle and lower lobes. It was not possible to access the middle lobe with the bronchoscope. With EBUS-TBNA, samples were taken from stations 7 and 4R. Findings from fine needle aspiration cytology (FNAC) were consistent with non-small-cell lung cancer and showed areas of necrosis.

Approximately two weeks after the EBUS-TBNA procedure, the patient presented to the emergency department with progressive dizziness, nausea and chest pain over several days. At this time, next-generation sequencing of the lung tumor had not been completed, and cancer treatment had not commenced.

On initial evaluation, the patient's heart rate was 99 beats per minute, blood pressure 94/79 mm Hg, respiratory rate 30 breaths per minute and the oxygen saturation 91% on ambient air. She was afebrile. Her electrocardiogram (ECG) showed no obvious abnormalities, but the baseline was variable and difficult to interpret.

**Fig. 1:**
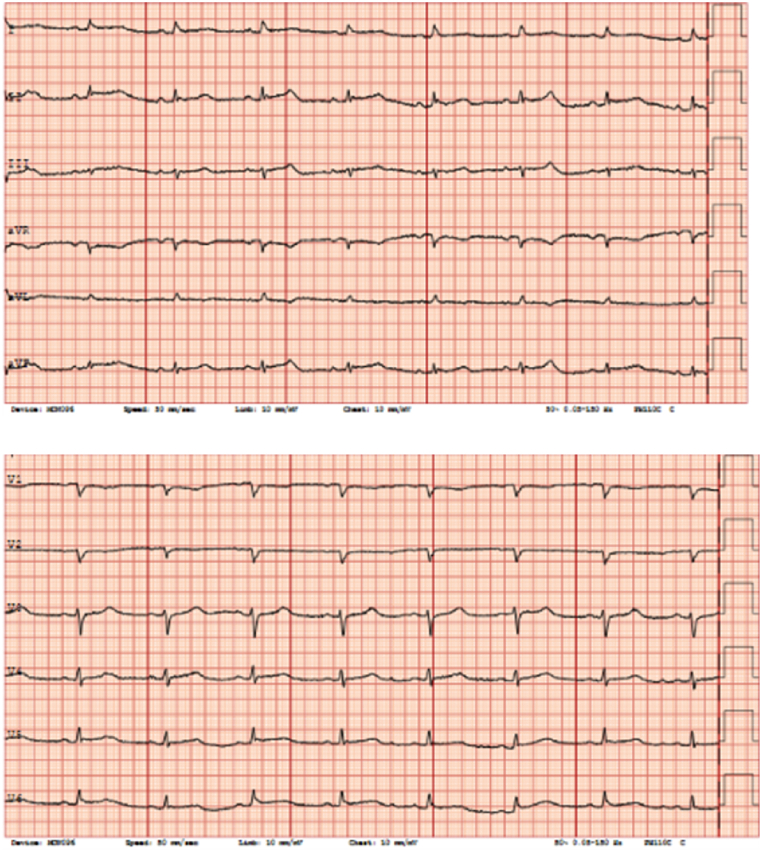
ECG of the patient after minimal exertion, showing ST-elevations in leads II, III and avF.

A minimal exertion (moving from a wheelchair to the hospital bed) led to dramatic changes in her vital signs and clinical status. She became pale, her chest pain worsened, and her blood pressure dropped. ECG showed ST-elevations in leads II, III, avF and V4-V6. (Fig. 1).

Within minutes, the patient went into cardiogenic shock with the lowest recorded blood pressure at 30/17 mm Hg. Blood tests were indicative of infection showing elevated leukocyte counts (25,9 x 10E9/L) and markedly raised C-reactive protein (CRP) level of 461 mg/L.

Echocardiography revealed a significant amount of pericardial effusion, consistent with cardiac tamponade. Subsequently, 650 ml of purulent fluid was drained from the pericardial space. Empirical antimicrobial treatment with Piperacillin/Tazobactam and Metronidazole was initiated based on a presumptive diagnosis of bacterial pericarditis. Her vital signs stabilized, and she showed clinical improvement.

Pericardial fluid and blood samples were sent for microbiological analysis. Culture of the pericardial fluid revealed growth of *Streptococcus anginosus,* a presumptively apathogenic *Neisseria species*, and several anaerobic bacteria including *Lancefieldella rimae, Prevotella pallens* as well as another *Prevotella species. Streptococcus anginosus* was also detected from blood culture and was susceptible to Penicillin, Cefotaxim and Clindamycin. Additionally, Polymerase chain reaction (PCR) from pericardial fluid identified DNA from *Streptococcus Intermedius* and *Fusobacterium necrophorum*. Streptococcus Intermedius was found to be sensitive to Penicillin and Clindamycin following resistance determination. Cytological examination of the pericardial fluid demonstrated a predominance of granulocytes, along with scattered macrophages and mesothelial cells. No malignant cells were detected.

**Fig. 2:**
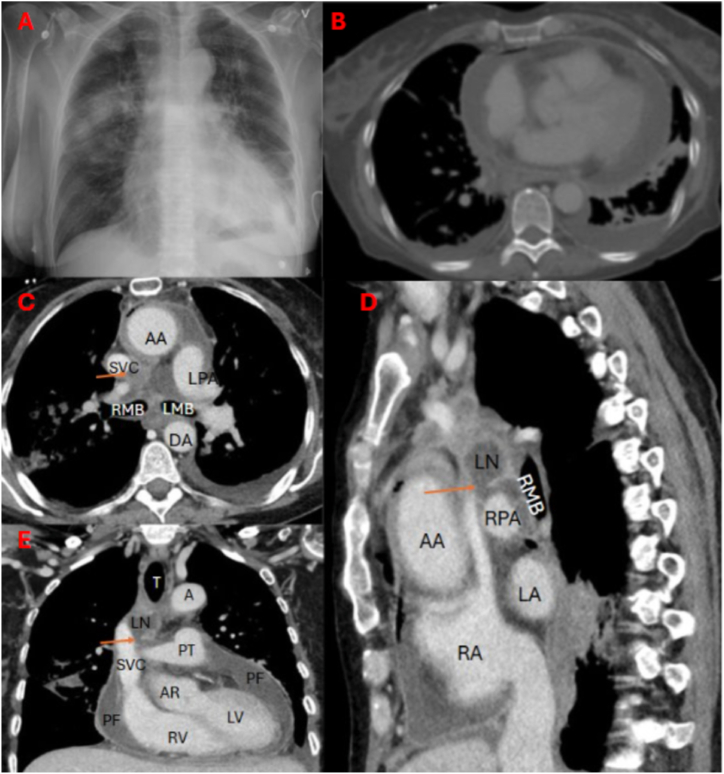
Chest CT scan (A), performed on the day of admission following pericardial drainage, shows bilateral upper lobe pulmonary opacities and cardiomegaly and thickened visceral and parietal pericardium (B). The trachea (T) is midline in position. The corresponding CT images provide axial (C), sagittal (D), and coronal (E) views of the chest, illustrating a defect within a necrotic pretracheal mediastinal lymph node (LN) at the level of the carina (indicated by an arrow), in relation to adjacent anatomical structures. The sagittal (D) and coronal (E) views further delineate the extent and anatomical context of the lesion, demonstrating a discontinuity in the lymph node wall, suggestive of a breach toward the adjacent pericardium. Labelled anatomical structures include: ascending aorta (AA), descending aorta (DA), superior vena cava (SVC), left pulmonary artery (LPA), right main bronchus (RMB), left main bronchus (LMB), trachea (T), aorta (A), pulmonary trunk (PT), pericardial fluid (PF), right atrium (RA), right ventricle (RV), left ventricle (LV), and right pulmonary artery (RPA). The coronal view (E) also highlights thickened visceral and parietal pericardial layers.

A chest X-ray and thoracic CT were performed on the same day (Fig. 2), revealing an enlarged cardiac shadow and bilateral upper lobe lung opacities. Communication between a necrotic mediastinal lymph node and the pericardial space was detected (Fig. 2C and D and E).

**Fig. 3:**
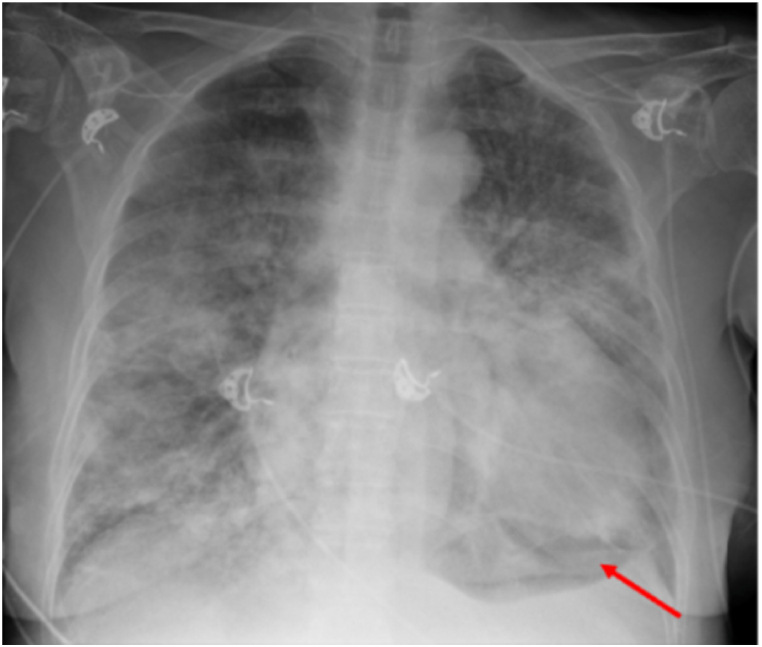
Chest X-ray performed two days after hospital admission and pericardial drainage shows an enlarged cardiac silhouette, congested pulmonary vasculature and bilateral pulmonary opacities. In addition, pneumopericardium is visible (arrow), likely secondary to the recent pericardial drainage.

Due to persisting hypoxemia, another X-ray was performed two days later, which revealed findings consistent with pulmonary edema and acute respiratory distress syndrome (ARDS) (Fig. 3).

The patient was treated with bi-level positive airway pressure (BiPAP) and high flow oxygen therapy. A repeat echocardiography performed two days later revealed an increase in pericardial effusion. Although she remained hemodynamically stable, additional drainage was deemed appropriate for infection control, and a pericardial drain was inserted for six days. During this period 750 ml of pericardial fluid was evacuated.

Cytological analysis of the fluid acquired during the second drainage detected reactive changes, with abundant granulocytes, scattered macrophages and reactive-appearing mesothelial cells. No malignant cells were identified.

The patient showed clinical and biochemical improvement after two days of BiPAP, intravenous antibiotics, and corticosteroids. Intravenous antibiotics were continued for two weeks followed by oral Metronidazole and Penicillin for an additional two weeks. She remained on corticosteroids for one month, including a tapering regimen.

Serial echocardiographies performed after discharge showed persistent pericardial effusion. However, since she was hemodynamically stable, no further drainage was undertaken. Treatment with intrapericardial fibrinolysis was considered but not initiated due to limited evidence and the presence of comorbidities.

Two weeks later, a follow up echocardiography revealed an organized hematoma lateral to the right ventricle, but no intervention was deemed necessary.

The first signs of constrictive pericarditis appeared on echocardiography four weeks later. Pericardiectomy was ruled out due to the patient's comorbidities, which made surgical intervention unlikely to be tolerated.

Subsequent follow-ups showed consistently improving cardiac function and only slightly reduced cardiac output. She also responded well to Osimertinib treatment for her non-small-cell lung cancer.

## 3 Discussion

This case illustrates inoculation of the pericardial cavity with *Fusobacterium necrophorum, Streptococcus intermedius*, *Streptococcus anginosus* and *Prevotella* during EBUS-TBNA, resulting in bacterial pericarditis in a patient with newly diagnosed lung cancer and subsequent development of constrictive pericarditis. The detection of Fusobacterium spp. suggests a polymicrobial infection, involving anaerobic bacteria [4].

Epidemiologic studies on acute pericarditis are limited, and its exact prevalence remains unknown. Most cases are believed to be of viral origin. Acute bacterial pericarditis is rare, occurring in less than 1% of hospitalized patients [5], and carries a high mortality rate of 30-50%, especially in cases complicated by cardiac tamponade. It typically arises as a secondary infection via contiguous spread.

Our case represents an exceptionally rare instance of purulent pericarditis following EBUS-TBNA, a procedure generally considered safe, with infectious pericarditis being a rare complication. A previous case report described two instances of bacterial pericarditis post EBUS-TBNA: one involving *Streptococcus viridans* which resulted in mortality, and another involving group *C beta-haemolytic Stretococcus* species [3]. Both cases involved sampling from station 4R (right lower paratracheal lymph node), a location adjacent to the superior pericardial recess.

In our case, *Streptococcus anginosus* was also isolated from the blood. Hematogenous spread after EBUS-TBNA is rare. In one study involving 45 patients, only three (7%) developed bacteriemia, with organisms including *Actinomycic spp, Streptococcus salivarius* and *Streptococcus mitis,* [6] all typically colonizing the oropharynx and none causing clinically significant infections [6].

In one study of 1045 patients, other infectious complications following EBUS procedures, such as pneumonia, intratumoral infection, lung abscess, pleuritis and empyema, have been reported in 4.4% of patients undergoing Endobronchial Ultrasound-guided Transbronchial Biopsy (EBUS-TBB) and not EBUS-TBNA. The most common pathogens were *Streptococcus* species and *normal flora*, but also included *Klebsiella* species, *Staphylococcus aureus, Escheria coli* and *Haemophilus influenzae* [7]. This study does not specify the proportion of cancer patients.

Another study reported a similar frequency of infectious complications following EBUS-TBNA [8]. Among 370 patients, 245 had suspected risk factors and 125 did not. Overall, 4.1% developed acute infectious complications. Identified risk factors included cavitation, lowdensity areas on CT, bronchial stenosis and necrotic tissue. The most common pathogens were *Pseudomonas aeruginosa* and *Streptococcus pneumoniae*. Necrosis and performing more than 10 needle passes were the strongest predictors of infection. In our study multiple punctures were performed (exact number unspecified), and necrosis was present, which may have contributed to bacterial inoculation of the pericardial cavity. Whether the patient's comorbid lung cancer increased susceptibility to post-EBUS-TBNA complications remains uncertain.

While needle contamination with oropharyngeal bacteria during TBNA is common, clinical infection is rare. In our case all detected microbes are part of the oral microbiota. Currently, there are no established guidelines or consensus on prophylactic antibiotic use before EBUS-TBNA. One Japanese study involving 484 patients undergoing EBUS-TBB found that among 251 patients who received prophylactic amoxicillin/clavulanate, the infection rate was 0.8%, compared to 5.2% in those who did not [9]. However, British Thoracic Society guidelines do not recommend prophylactic antibiotics prior to bronchoscopy.

Our patient also developed constrictive pericarditis, a rare complication of acute pericarditis. Studies have shown that incomplete drainage of purulent pericarditis increases the risk of constriction [10]. One prospective cohort study of 500 cases found that constrictive pericarditis occurred in 1.8% of viral/idiopathic cases, compared to 8.3% in non-viral/non-idiopathic cases [11]. Bacterial pericarditis had the highest incidence, with 52.74 cases per 1000 person-years, versus 0.76 for viral/idiopathic pericarditis. In our case, bacterial etiology and incomplete drainage likely contributed to the development of constrictive pericarditis.

## 4 Conclusion

Bacterial pericarditis following EBUS-TBNA is a rare but serious complication and appears to be associated with identifiable risk factors. In our patient the necrosis within the sampled lymph node and its proximity to the superior pericardial recess were likely contributors. This case highlights the need for caution when performing EBUS-TBNA in patients with necrotic lesions near the pericardium.

## CRediT authorship contribution statement

**Rachna S. Gulati:** Writing – original draft, Writing – review & editing, Formal analysis, Project administration. **Håkon Kravdal:** Data curation. **Heidi Hoven:** Data curation. **Erlend Eriksen:** Data curation. **Hans Kristian Haugland:** Data curation. **Tehmina Mustafa:** Supervision, Validation, Writing – review & editing.

## Ethics declarations

Written informed consent was obtained from the patient for publication of this case report and any accompanying images. Copies of the written consent are available for review.

## Declaration of competing interest

The authors declare that they have no known competing financial interests or personal relationships that could have appeared to influence the work reported in this paper.
